# Detection of *Leptosphaeria maculans* and *Leptosphaeria biglobosa* Causing Blackleg Disease in Canola from Canadian Canola Seed Lots and Dockage

**DOI:** 10.3390/plants5010012

**Published:** 2016-03-01

**Authors:** W. G. Dilantha Fernando, Xuehua Zhang, Chami C. Amarasinghe

**Affiliations:** Department of Plant Science, University of Manitoba, Winnipeg, MB R3T 2N2, Canada; zxh2012xuehua@gmail.com (X.Z.); umamarac@myumanitoba.ca (C.C.A.)

**Keywords:** Blackleg, *Leptosphaeria maculans*, *Leptosphaeria biglobosa*, canola, dockage, seed lots, admixture

## Abstract

Blackleg, caused by *Leptosphaeria maculans*, is a major threat to canola production in Canada. With the exception of China, *L*. *maculans* is present in areas around the world where cruciferous crops are grown. The pathogen can cause trade barriers in international canola seed export due to its potential risk as a seed contaminant. The most recent example is China restricting canola seeds imported from Canada and Australia in 2009. Therefore, it is important to assess the level of Blackleg infection in Canadian canola seed lots and dockage (seeds and admixture). In this study, canola seed lots and dockage samples collected from Western Canada were tested for the presence of the aggressive *L*. *maculans* and the less aggressive *L*. *biglobosa*. Results showed that both *L. maculans* and *L. biglobosa* were present in seed lots and dockage samples, with *L. biglobosa* being predominant in infected seeds. Admixture separated from dockage had higher levels of *L. maculans* and *L. biglobosa* infection than samples from seed lots. Admixture appears to harbour higher levels of *L*. *maculans* infection compared to seeds and is more likely to be a major source of inoculum for the spread of the disease than infected seeds.

## 1. Introduction

Blackleg, also known as phoma stem canker, is an economically important disease of *Brassica napus* (canola, oilseed rape) and many other cruciferous species worldwide. Blackleg epidemics occur regularly in oilseed rape crops and have been observed in Europe, Canada, and Australia [1]. On oilseed rape, it causes serious losses in Europe, Australia, and North America; worldwide losses are estimated at >$900 million per growing season [1,2]. Two species, aggressive *Leptosphaeria maculans* and non-aggressive (less aggressive) *L. biglobosa* can cause Blackleg disease on canola [3,4,5]. These species co-exist in most *B. napus* growing areas of the world [3,4,5] except in China where only *L. biglobosa* has been reported [1,6,7,8,9,10]. The taxonomic history of *L. maculans* has been relatively unclear until recently. The first common distinction was made between highly-virulent or aggressive “A” group strains (currently referred to as *L. maculans*) that produced the phytotoxin sirodesmin PL and were also able to infect canola, and the weakly-virulent or non-aggressive, “B” group strains (currently referred to as *L. biglobosa)* that did not produce sirodesmin PL nor could infect canola [11]. *Leptosphaeria maculans* survives from season to season in residues after harvest and ambient weather provides conditions favorable for the initiation, development, and maturation of pycnidia and pseudothecia. Ascospores and pycnidiospores released from infected residues serve as a source of primary inoculum for infection of canola seedlings [4,12]. Although *L. maculans* is more aggressive than *L. biglobosa*, their life cycles are similar. All parts of the susceptible canola/oilseed rape host can be colonized by these pathogens, including the root, stem (mainly the base), upper stem parts, leaves, cotyledons, and seeds [13]. It has been reported that the pathogens causing Blackleg are adaptable, since the disease is very damaging on both spring and winter (autumn-sown) types of oilseed rape in a wide range of climates [4]. Therefore, it is possible that *L. maculans* “brassicae” has successfully spread into new areas by global trade and/or natural dispersal [4].

To date, Blackleg has not been a serious problem in China and only the less aggressive *L. biglobosa* has been found there [1,6,7,8,9,10]. In China winter oilseed rape is often followed by several weeks of flooding with a paddy rice crop [14]. It has been reported that flooding can rapidly reduce the survival of the Blackleg pathogen in canola stubble [15]. *L. maculans* is considered a global invasive species, it has recently spread across Canada (1975–1998), from the USA into Mexico (2001) and eastwards across Europe into Poland (1994–2007), where only the less damaging *L. biglobosa* was previously present [1]. Therefore, there may be a potential risk of spreading this pathogen into China via global canola seed trade.

Blackleg is the most important fungal disease of canola in Western Canada. In the last two decades, the disease has been managed mainly using resistant cultivars and longer crop rotations. However, in the recent past, tighter rotations driven by market demand have created situations favorable for increased risk of disease outbreaks and resistance breakdown [16,17]. Currently *L. maculans* is listed as a quarantine disease in China. Since 1994, China imports several million tons of canola from Canada, where *L. maculans* is a prevalent pathogen on canola [4]. In 2009, the Chinese government restricted the importation of oilseed rape seeds from Canada [10]. The potential risk was considered to be the import of *L. maculans*-infected canola seed lots, dockage, and the associated admixture to *L. maculans* free canola regions/fields. The associated admixture includes small pieces of crop debris and parts from pod sheath and branches, *etc.* This resulted in a decrease in exports of oilseed rape seeds from Canada in 2010 and 2011. However, by 2011, China eventually agreed to allow access to several canola oil crushing-plants at or near ports and not in canola growing regions. Therefore, the potential risk of introducing the pathogen through infected seeds and dockage needs to be determined in order to maintain continuous access to the Chinese canola market.

Due to the importance of this disease in global trade, this research investigated canola seed lots and dockage infection by the two *Leptosphaeria* species in Canada. The objective of the study was to determine the relative risk that canola seed and dockage play in introducing *L. maculans* to areas devoid of this pathogen. To understand this better, the following areas were investigated, (1) the frequency and viability of blackleg infection on seed lots and dockage, and (2) the ability of infected seed lots and dockage to produce *L. maculans* primary inoculum.

## 2. Results

### 2.1. Frequency and Viability of Leptosphaeria Species in Seed Lots Samples

**Figure 1:**
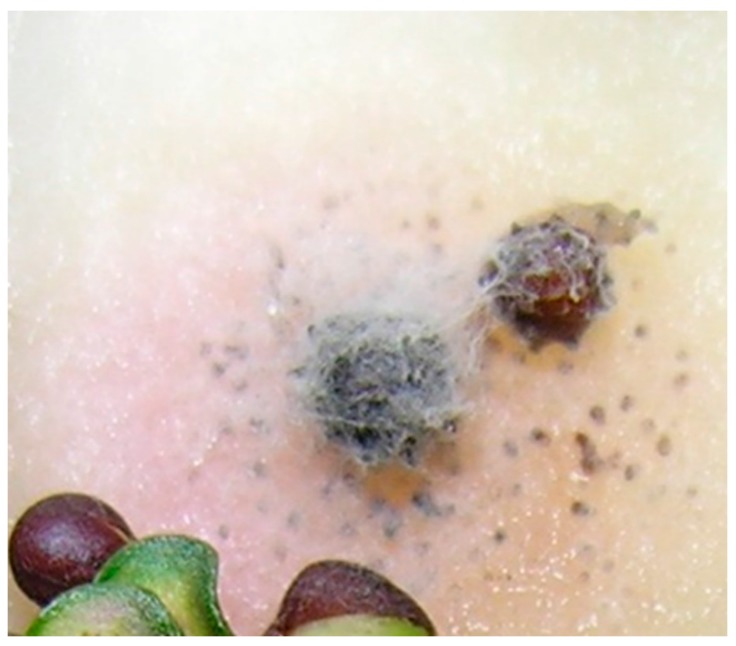
Canola seeds infected with *Leptosphaeria* species. Dark dots on and around seeds represents pycnidia.

**Figure 2:**
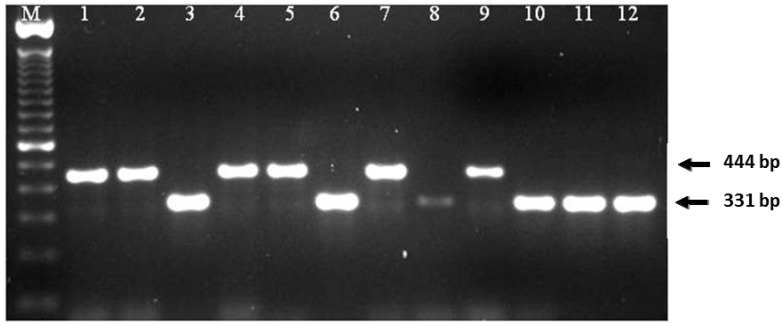
Blackleg isolates identified using species-specific polymerase chain reaction (PCR) assay. Lanes: M-100bp DNA ladder; 1, 2, 4, 5, 7, 9––*Leptosphaeria biglobosa* “brassicae” (444 bp); 3, 6, 8, 10, 11, 12––*Leptosphaeria maculans* “brassicae” (331 bp).

**Figure 3:**
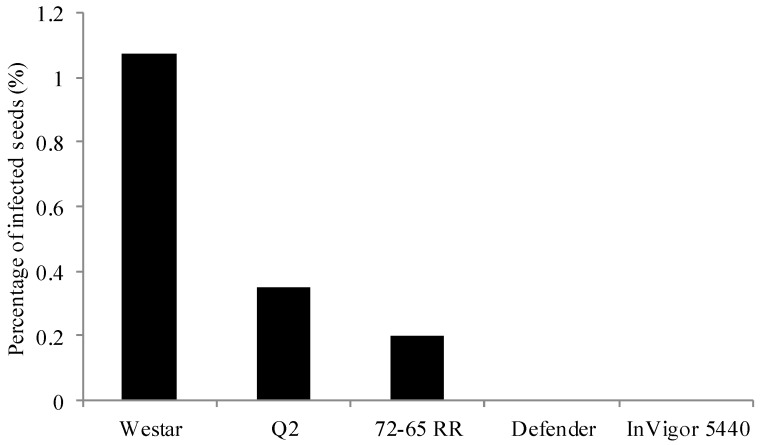
Percentage of *Leptosphaeria maculans* infected seeds in canola samples with different level of Blackleg resistance. Level of resistance: S––susceptible; MS––moderately susceptible; R––resistant; MR––moderately resistant. Westar: S; Q2: MS; 72-65RR: R; Defender: MR; InVigor 5440: R.

Twenty-five seed lot samples collected from co-op sites were incubated in moisture chambers (1000 seeds per sample, 25,000 individual seeds) to detect Blackleg infection in canola seeds. All seeds that showed formation of pycnidia or hyphae were considered infected (Figure 1). Out of 25 samples, five samples did not show any seed infection. A total of 1072 blackleg isolates were isolated from infected seeds, and cultured on V8^®^ agar plates to obtain pycnidiospores for DNA extraction. To identify *L. maculans* “brassicae” and *L. biglobosa* “brassicae”, species-specific primers described by Liu *et al.* [18] was adopted in this study. These primers are developed based on the sequence differences of the ribosomal RNA region between these two species. In the PCR assay, *L. maculans* “brassicae” gave an amplified product of 331 bp while *L. biglobosa* “brassicae” gave 444 bp of amplified product (Figure 2). Ten of each amplified products were sequenced to confirm the accuracy of the PCR amplified products. Out of all 1072 isolates, only 61 (5.7%) isolates were identified as *L. maculans* “brassicae” and 1011 (94.3%) were *L. biglobosa* “brassicae” (Table 1). Therefore, when considering a single seed, the possibility of having the *L. maculans* infection was 0.24% and *L. biglobosa* was 4.04%. The data showed that canola varieties rated as susceptible to Blackleg, such as Westar, had higher levels of *L. maculans* seed infection (1.07%, *i.e*., 43 *L. maculans* infected seeds among 4000 tested Westar seeds from four different sites) compared to varieties rated as resistant, such as Q2 (0.35%), 72-65RR (0.2%), and Invigor 5440 (0%) (Figure 3). The highest number of *L. mauclans* infected seeds (30) was identified in Westar samples collected from Plum Coulee, MB, while the highest number of *L. biglobosa*-infected seeds (300) was found in seed samples collected from Canora, SK, Canada. All infected seeds produced pycnidia. Some of the infected seeds could germinate and develop into infected cotyledons; however, all of these cotyledons died without any further growth. In summary, *L. biglobosa* is the predominant species present in infected canola seeds.

### 2.2. Identification of Leptosphaeria Species in Dockage 

A total of 38 canola dockage samples (composed of seeds, chaff and straw) were collected from commercial fields or elevators to further identify Blackleg infection in seeds and the associated admixture. Seeds were first separated from dockage and seed infection analysis was performed as mentioned above (Section 2.1). The remaining chaff and straw were considered as admixture. DNA was extracted from admixture (a total of 100 individual DNA samples per admixture sample) to dectect Blackleg infection in admixture using species-specific PCR assay [18]. Comparison of Blackleg infection in seed and admixture samples is shown in Table 2. Of the 38 samples tested, *L. maculans* seed infection was observed in 9 and *L. biglobosa* in 14 samples. The level of seed infection of the above samples ranged 0%–0.4% for *L. maculans* and 0%–0.2% for *L. biglobosa*. Higher levels of Blackleg infection in admixture was observed in 14 samples for *L. maculans* and nine samples for *L. biglobosa*, ranging 0%–31% and 0%–10%, respectively. Seed infection with both species was observed in seven samples, and admixture infection with both species was observed only in two samples. Among 38 samples, 22 samples did not show any seed infection and 18 samples did not show any admixture infection either by *L. maculans* or *L. biglobosa*. Taken together, these data indicated that admixture carried higher level of blackleg infection, mainly *L. maculans* than seeds.

### 2.3. Ability of Admixture to Produce Blackleg Inoculum

To understand the ability of admixture to produce Blackleg inoculum, admixture samples were incubated in moisture chambers at different temperatures. No pycnidia was observed on admixture kept at 5 °C and 15 °C. Admixture samples at room temperature (25 °C) showed some pycnidia growth, although the typical amethyst ooze was not visible. Sexual fruiting bodies (pseudothecia) were not observed on infected seeds as environmental conditions within the moisture chamber may not be favourable for sexual reproduction. Some of those pycnidia were excised and viewed under the microscope to confirm the presence of spores. Pycnidiospores were abundantly present in all of the samples examined. Out of 15 pieces tested individually, nine had *L. maculans*; but *L. biglobosa* was not detected in any of the samples. The above results suggested that admixture can produce Blackleg inoculum under favourable conditions, and environmental factors, such as temperature and relative humidity, may affect the ability of admixture to produce Blackleg inoculum.

### 2.4. The Ability of Admixture to Cause Infection

**Figure 4:**
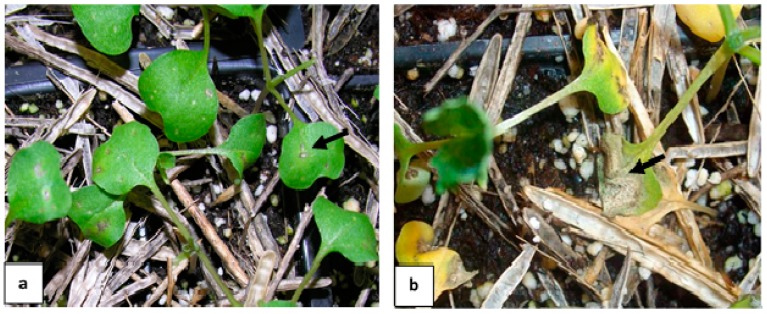
Reactions of Westar seedlings to inoculum produced by admixture. Arrows indicate (**a**) lesions starting to form seven days after wounding, and (**b**) advanced tissue collapse accompanied by pycnidia formation 14 days after wounding.

Greenhouse assays were performed to detect the ability of admixture to cause infection on canola seedlings. Seven days after wounding, infections were visible on some Westar cotyledons from trays with admixture (Figure 4a). No cotyledon infection was observed in control plants. After two weeks, infections had progressed in more than 50% of plants and they exhibited different levels of infection (Figure 4b). Pycnidia were visible on many infected canola cotyledons. An average of 37% of the infection sites was considered susceptible (rating scale of 0–9, rating ≥5.0). These results indicated that admixture with blackleg infection can cause seedling infection.

## 3. Discussion

Canola is one of the major cash crops in Canada generating a substantial annual turnover. Diseases such as Blackleg, can cause significant losses unless managed properly. Blackleg of canola is mainly managed in Canada through the use of resistant varieties, fungicides, and cultural practices [16,19]. In addition to crop losses, Blackleg can have an impact on accessing foreign markets, especially in areas where the pathogen has not been reported before. A bulk of Canadian canola seeds are exported to China for extraction of oil. Chen *et al*. [20] identified *L. maculans* from imported canola seeds in China. Due to the possible risk of introduction of this pathogen through imported seeds, there is a restricted market access for Canadian canola in China. In view of this, research was mandated to assess the level of Blackleg infection in Canadian seed lots and dockage samples.

The results from this study showed that although both species of the pathogen could be found in canola seeds, the most frequent one was the less aggressive *L. biglobosa* (0%–0.4%). The *L*. *maculans* levels were very low, ranging 0%–0.2%. Chigogora and Hall [21] had reported seed infection levels up to 5.1% in Canadian canola, which were much higher than levels reported in previous studies [22,23]. These elevated seed infection levels were attributed to weather conditions, availability of inoculum in the season and increase of Blackleg severity and incidence [16,21]. It is worth noting that higher frequencies of *L. maculans* were observed in samples collected from Plum Coulee (30 isolates) and Vegreville (23 isolates). These are the areas where more virulent *L. maculans* isolates were identified and reported [24].

Differences between varieties were observed in this study. For example, in Plum Coulee, Westar, a susceptible variety that is no longer grown commercially had a higher percentage of *L*. *maculans* seed infection (3%) compared to the moderately resistant variety Defender which had no *L. maculans* seed infection. In addition, other commercial varieties showed low levels of *L*. *maculans* seed infection. This could indicate that inherent Blackleg resistance in canola varieties plays a major role in lowering the risk of seed infection levels, even in areas where pathogen population is relatively high.

Infected seeds mostly rotted before they could germinate. The few seeds that germinated did not grow into seedlings, indicating that infected seeds may not grow into infected plants and produce additional inoculum. McGee [22] reported that inoculum from plant residue was contributing more to seedling and adult plant infections than seed infection. Barbetti and Khangura [25] also reported that infected seeds are often shrivelled and might not germinate. Seed lots with low percentages of infected seeds were observed to produce cotyledons with low infection rate [26]. Moreover, Van de Wouw *et al.* [27] reported that viability of *L. maculans* in infected seeds reduced over time. Although the potential risk of introducing the pathogen in to previously non-infested areas via infected seeds cannot be ignored, the risk it poses is minimal [21]. In China, imported canola seeds are solely used for crushing, the risk of spreading blackleg disease via imported seeds is presumably low. In addition, *L. biglobosa,* the less aggressive species which already has been found in China was more prevalent in infected seeds. Although in some studies conducted in Europe indicated that Chinese *B. napus* cultivars were susceptible to *L. maculans*, recent studies have shown that high percentage of *B. napus* cultivars/lines from China showed both major gene resistance and adult plant resistance [1,16,28]. This shows that Chinese *B. napus* cultivars have the necessary resistance mechanism to withstand the potential risk of *L. maculans* if it is introduced via infected seeds. However, we still cannot fully ignore the potential threat that it may pose by introduction of *L. maculans* into areas where it has not been previously reported.

According to this study, all infected seeds and admixture produced pycnidiospores. Pycnidiospores have been reported to be the primary sources of inoculum in Western Canada rather than ascospores [12,29]. *L. maculans* was present in admixture in higher percentages compared to seed infection. Our data support the assumption by Zhang *et al.* [11] that admixture was likely to be more important than infected seeds in transmitting *L. maculans*. It was also evident that the pathogen was viable on admixture and could infect cotyledons. Hence, admixture appears to be a more important component in terms of carrying significant levels of inoculum to introduce the disease into new areas than infected seeds. Therefore, this study recommends the efficient removal and management of admixture, especially at crushing sites, to significantly minimize the risk of introduction of Blackleg pathogens to new areas either via dockage or seed lots.

## 4. Materials and Methods

### 4.1. Sample Collection

Seed lots samples were collected from co-op sites in 2011, while dockage samples were collected from commercially-grown canola fields and/or from elevators in 2012. Samples were transported to the laboratory in paper or plastic bags and stored at room temperature (25 °C) until processing (all samples were processed within three months after collection). Blackleg resistance of canola varieties from fields was evaluated using a rating scale of 0–5 based on internal infection of the stem base (Western Canada Canola/Rapeseed Recommending Committee). At least 100 stubbles per field were evaluated for Blackleg resistance. The average rating score (ARS) was transformed into level of resistance. Level of resistance: S––susceptible; MS––moderately susceptible; R––resistant; MR––moderately resistant.

### 4.2. Identification of Leptosphaeria Species from Seed Samples

A total of 25 seed samples were used for seed infection analysis. The approach traditionally used to determine the severity of seed contamination is to plate surface sterilized seed on agar and record the number of seeds that produce colonies of the pathogen(s). This method is accepted by the International Seed Testing Association (ISTA) as a means of determining the incidence of pathogen infection in seed [30]. Since we were more interested in examining the internal fungal infection of canola seeds, a similar approach was used as described by Kharbanda and Stevens [31]. At first, 1000 seeds per sample were surface disinfected in 5% sodium hypochlorite (NaOCl) for 1 min, followed by rinsing with sterile distilled water three times. Seeds were air dried and transferred onto several layers of moistened sterile paper towels in a sterile moist chamber to absorb moisture for 24 h, followed by 24 h at 4 °C to slow down germination. Seeds were then kept in the moist chamber at room temperature for five days while monitoring fungal growth. Seeds showing fungal growth were placed on V8^®^ juice agar (200 mL of V8^®^ juice, 0.75 g of CaCO_3_, 15 g of agar, 800 mL of distilled H_2_O) plates amended with streptomycin sulphate (0.35% (*w*/*v*)). All seeds that showed formation of pycnidia or hyphae on or around them were considered infected. Seeds that did not exhibit pycnidia or hyphae were considered non-infected. Pure cultures of Blackleg isolates were made by single pycnidiospore isolation and all isolates were preserved until further use.

Isolates were then identified as *L. maculans* or *L. biglobosa* by species-specific polymerase chain reaction (PCR) assay. DNA was extracted from pycnidiospores according to the method described by Lee and Taylor [32] with the following modification. Spores were initially homogenised using 0.2 mm ceramic beads for 45 sec at 6500 rpm in a Precellys^®^ 24 homogeniser (Bertin Technologies, Montigny-le-Bretonneux, France). PCR assay using species-specific primers described by Liu *et al.* [18] was adopted in this study to determine the identity of each isolate as either *L. maculans* “brassicae” or *L. biglobosa* “brassicae”. Following primers were used in the PCR assays; LbigF 5ʹ-ATCAGGGGATTGGTGTCAGCAGTTGA-3ʹ, LmacF 5ʹ-CTTGCCCACCAATTGG-ATCCCCTA-3ʹ, and a common reverse primer LmacR 5ʹ-GCAAAATGTGCTGCGCTCCAGG-3ʹ [18]. The PCR amplified product from *L. maculans* “brassicae” gave a 331 bp amplified product while *L. biglobosa* “brassicae” gave a 444 bp of amplified product.

To determine whether Blackleg-infected seeds can develop disease in canola cotyledons, infected seeds that were able to germinate were transplanted into soilless potting mix. These pots were placed in a controlled growth chamber at 16 °C (night) and 21 °C (day), with a 16 h photoperiod.

### 4.3. Determination of Leptosphaeria Species in Dockage Samples Collected from Commercial Fields

Canola dockage samples collected were composed of seeds, chaff, and straw. A total of 38 dockage samples were collected for the analysis. At first, seeds were separated from dockage. The remaining chaff and straw from dockage samples were considered as admixture, theses include small pieces of crop debris such as sections of the pod sheath and branches. To determine the level of Blackleg infection in admixture, DNA was extracted from 100 randomly selected single chaff/straw pieces (one DNA sample per single chaff/straw piece) from each of the commercial samples separately as described by Demeke *et al*. [33]. Identification of *Leptosphaeria* species as *L. maculans* or *L. biglobosa* was done using species-specific primers described by Liu *et al*. [18]. In order to compare the levels of infection between seeds and admixture from the same samples, seeds separated from dockage were tested for Blackleg infection as described above.

### 4.4. The Ability of Admixture to Produce Primary Inoculum

An experiment was carried out to investigate the ability of admixture to produce primary inoculum such as pycnidiospores or ascospores. Admixture samples were kept in a moist chamber (plastic box lined with fine sand and paper towel) at 5 °C, 15 °C, and ambient temperature (25 °C). Samples were monitored every day for three weeks for the appearance of pycnidia. The identity of those pycnidia as *L. maculans* or *L. biglobosa* was determined by PCR assay [18].

### 4.5. The Ability of Admixture to Cause Infection

A universal blackleg susceptible canola variety, Westar was used to test the ability of admixture to cause plant infection. The seedlings (covered with admixture from Westar dockage samples) were grown in trays filled with Sunshine mix #4 (SunGro Horticulture, Agawam, MA, USA) in a controlled growth chamber (same growth condition as described in Section 4.2). Westar plants were grown in three replicates, each replicate having 32 plants. A control was performed without adding admixture. To create route of infection, cotyledons were wounded at multiple sites (ten wounds per plant) seven days after seeding. A transparent plastic cover was placed over tray to increase humidity. The appearance and progression of blackleg infection was monitored for 14 days after wounding. Infected cotyledons were rated based on a 0 to 9 scale where 0 = no infection and 9 = tissue collapse and appearance of sporulation [34]. Ratings ≥5.0 were considered virulent and ≤4.9 were considered avirulent.

## 5. Conclusions

Both *L. maculans* and *L. biglobosa* were present in seed and dockage samples collected from Western Canada, whereas the majority of seed infection was caused by *L. biglobosa*.Compared with seed lots, admixture separated from dockage appears to harbour higher levels of *L. maculans* and *L. biglobosa* infection, with *L. maculans* infection being predominant. Admixture is able to produce primary inoculum (pycnidiospores) and cause severe seedling infection on Westar. Thus, admixture is more likely to be a major source of inoculum than infected seeds during the spread of the disease. It is therefore essential to efficiently remove admixture, especiallyat crushing sites, to minimize the risk of introducing *L. maculans* to new areas.

## Figures and Tables

**Table 1:** Summary of Blackleg isolates recovered and identified from canola seed lots collected from co-op sites across Western Canada in 2011.

Sample	Location	Level of Resistance ^a^	Number of Isolates ^b^		
			*L. maculans*	*L. biglobosa*	Total
Westar	Vegreville, AB	S	13	39	52
Q2 – Field 1 **^c^**	Vegreville, AB	MS	7	39	46
Q2 – Field 2 **^c^**	Vegreville, AB	MS	0	41	41
Defender	Vegreville, AB	MR	2	21	23
72-65 RR	Lloydminster, AB	R	4	44	48
Defender	Carman, MB	MR	0	3	3
Westar	Carman, MB	S	0	0	0
Defender	Plum Coulee, MB	MR	0	47	47
Westar	Plum Coulee, MB	S	30	132	162
Defender	Roland, MB	MR	0	3	3
Westar	Roland, MB	S	0	3	3
45H29	Brandon, MB	R	0	0	8
72-65 RR	Brandon, MB	R	0	0	0
CDS10-053	Brandon, MB	N/A	0	0	0
InVigor 5440	Brandon, MB	R	0	0	0
InVigor 5440	Brandon, MB	R	0	12	12
Defender	N. Battleford, SK	MR	0	77	77
SC2	Canora, SK	N/A	0	300	300
SC7	St Brieux, SK	N/A	1	13	14
SC9-1	Pelly, SK	N/A	0	1	1
SC9-2	Pelly, SK	N/A	2	30	32
CR47	Springside, SK	N/A	0	3	3
Defender	N. Battleford, SK	MR	2	87	89
SC1	Canora, SK	N/A	0	116	116
BHSW2	Birch Hills, SK	N/A	0	0	0
			61	1011	1072

^a^ Blackleg resistance of canola varieties in some fields was evaluated using a rating scale of 0–5 based on internal infection on the cross-section of the stem base (Western Canada Canola/Rapeseed Recommending Committee). At least 100 stubbles per field were evaluated for Blackleg resistance. The average rating score (ARS) was transformed into level of resistance. Level of resistance: S––susceptible; MS––moderately susceptible; R––resistant; MR––moderately resistant; N/A––not available; ^b^ A total of 1000 seeds per sample were analyzed for seed infection; ^c^ Q2 seed samples were collected from different fields in Vegreville, AB, Canada.

**Table 2:** Seed and admixture infection of commercially grown canola dockage samples across Western Canada in 2012.

Sample	Location	Level of Resistance ^a^	Seed Infection (%) ^b^		Admixture Infection (%) ^c^	
			*L. maculans*	*L. biglobosa*	*L. maculans*	*L. biglobosa*
L150	North Battleford, SK	R	0.2	0.2	10	0
73-45 RR	Lloydminster, SK	R	0.2	0.2	10	2
73-45 RR	Speers, SK	R	0.1	0.1	6	0
45H29	Lloydminster, SK	R	0.1	0.1	10	6
73-45 RR	Marshall, SK	R	0.1	0.1	0	2
45S52	Speers, SK	MR	0.2	0.2	8	0
9553	Lloydminster, SK	R	0.4	0.2	2	0
43E02	Grand Prairie, AB	MR	0	0	0	0
73-45 RR	Rycroft, AB	R	0	0	2	0
1012 RR	Girouxville, AB	R	0	0	0	2
5440	Neepawa, MB	R	0	0.1	0	2
L130	Griswold, MB	R	0	0.1	31	0
Nexera	Justice, MB	N/A	0	0	0	0
5440	Neepawa, MB	R	0	0	17	0
73-45	Oak River, MB	N/A	0.1	0	0	0
5440	Dauphine, MB	N/A	0.1	0	13	0
HEAR	Souris, MB	N/A	0	0	8	0
INVIGOR	Minnedosa, MB	N/A	0	0	2	0
D	Duff, SK	N/A	0	0	0	0
D2	Duff, SK	N/A	0	0	0	0
71-45	St. Paul, AB	MR	0	0	0	0
INV5020	Mallaig, AB	R	0	0.1	0	0
6040 RR	Lloydminster, SK	R	0	0	0	2
1012 RR	Douglas, MB	R	0	0	0	2
1841 RR	Lloydminster, SK	R	0	0	0	2
INV5440	Justice, MB	R	0	0	0	0
INV5020	Mallaig, AB	R	0	0	12	0
INV5020	AB	R	0	0.1	0	0
INV5440	AB	R	0	0	0	0
6060 RR	Justice, MB	R	0	0.2	0	0
CI-Nexera	Justice, MB	-	0	0.1	0	0
5440	St. Benedict, SK	R	0	0.1	0	0
45H29	Cudworth, SK	R	0	0	0	0
72-65	St. Benedict, SK	R	0	0	0	0
K	AB	N/A	0	0	0	0
L	AB	N/A	0	0	0	0
M	AB	N/A	0	0	0	0
Cargill 4	SK	N/A	0	0	10	10

^a^ Blackleg resistance of canola varieties in some fields was evaluated using a rating scale of 0–5 based on internal infection on the cross-section of the stem base (Western Canada Canola/ Rapeseed Recommending Committee). Blackleg rating: S––susceptible; MS––moderately susceptible; R––resistant; MR––moderately resistant; N/A––not available. At least 100 stubbles per field were evaluated for Blackleg resistance. The average rating score (ARS) was transformed into level of resistance; ^b^ A total of 1000 seeds per site were analyzed for seed infection; ^c^ A total of 100 individual DNA samples per admixture sample were analyzed.
